# Infection–nutrition feedbacks: fat supports pathogen clearance but pathogens reduce fat in a wild mammal

**DOI:** 10.1098/rspb.2024.0636

**Published:** 2024-07-17

**Authors:** Rachel A. Smiley, Brittany L. Wagler, William H. Edwards, Jessica Jennings-Gaines, Katie Luukkonen, Kara Robbins, Marguerite Johnson, Alyson B. Courtemanch, Tony W. Mong, Daryl Lutz, Doug McWhirter, Jennifer L. Malmberg, Blake Lowrey, Kevin L. Monteith

**Affiliations:** ^1^ Haub School of the Environment and Natural Resources, 804 E Fremont Street, Laramie, WY 82071, USA; ^2^ Department of Zoology and Physiology, Cooperative Fish and Wildlife Research Unit, University of Wyoming, 1000 University Avenue, Laramie, WY 82071, USA; ^3^ Department of Wyoming Game and Fish, Wildlife Health Laboratory,1174 Snowy Range Road, Laramie, WY 82072, USA; ^4^ Department of Wyoming Game and Fish, 420 N Cache Street, Jackson, WY 83001, USA; ^5^ Department of Wyoming Game and Fish, 2820 WY-120, Cody, WY 82414, USA; ^6^ Department of Wyoming Game and Fish, 260 Buena Vista Drive, Lander, WY 82520, USA; ^7^ Department of Veterinary Sciences, University of Wyoming, 1174 Snowy Range Road, Laramie, WY 82070, USA; ^8^ US Geological Survey, Northern Rocky Mountain Science Center, 2327 University Way, Bozeman, MT 59715, USA

**Keywords:** nutritional condition, host–pathogen interactions, *Ovis canadensis*, sublethal effects

## Abstract

Though far less obvious than direct effects (clinical disease or mortality), the indirect influences of pathogens are difficult to estimate but may hold fitness consequences. Here, we disentangle the directional relationships between infection and energetic reserves, evaluating the hypotheses that energetic reserves influence infection status of the host and that infection elicits costs to energetic reserves. Using repeated measures of fat reserves and infection status in individual bighorn sheep (*Ovis canadensis*) in the Greater Yellowstone Ecosystem, we documented that fat influenced ability to clear pathogens (*Mycoplasma ovipneumoniae*) and infection with respiratory pathogens was costly to fat reserves. Costs of infection approached, and in some instances exceeded, costs of rearing offspring to independence in terms of reductions to fat reserves. Fat influenced probability of clearing pathogens, pregnancy and over-winter survival; from an energetic perspective, an animal could survive for up to 23 days on the amount of fat that was lost to high levels of infection. Cost of pathogens may amplify trade-offs between reproduction and survival. In the absence of an active outbreak, the influence of resident pathogens often is overlooked. Nevertheless, the energetic burden of pathogens likely has consequences for fitness and population dynamics, especially when food resources are insufficient.

## 1. Introduction

The impacts of disease on wildlife populations are most apparent during mass-mortality events; however, after initial outbreaks subside, pathogens responsible for the epizootic often become enzootic in populations, and their effects become indirect and insidious [1]. The effects of enzootic pathogens when not causing clinical disease often are difficult to detect, and thus overlooked, but can have measurable consequences for populations [2]. Further, pathogens and nutritional condition of the host can interact; periods of nutritional stress can exacerbate consequences of pathogens on host demographic rates [3,4] and contribute to increased infection rates or subsequent outbreaks [5,6]. Despite the fundamental role that interplay between pathogens and host energetics can have on animal fitness and population dynamics, the indirect costs of enzootic pathogens remain underappreciated [2].

The energetic income and reserves that wild animals use throughout the year underpins their reproductive success, survival and ultimately fitness [7–9]. Insufficient energetic reserves (often fat) can limit reproductive success, and even come at the cost of survival when resources are scarce. In temperate ungulates, which are finely tuned to their environments, small changes in body fat can result in substantial changes to fitness components [7,10,11]. For example, a 10 percentage point difference in body fat in autumn (i.e. from 20% to 10% body fat) reduces the probability of survival by over 60% during a severe winter for mule deer (*Odocoileus hemionus*) [12]. In bighorn sheep (*Ovis canadensis*), probability of pregnancy decreases from over 90% to less than 50% with less than a 4 percentage point decrease in body fat (i.e. from 7.7% to 4%) [13]. Moreover, lack of energetic reserves may increase susceptibility to infection [14,15] because nutritional deficiencies can suppress immune function [16,17]. If interplays between pathogens and energetics are additive, and infection leads to reduced condition that exacerbates vulnerability to further infection [15], pathogens may cause a cyclical decline of host condition, reproductive success and survival. Thus, populations experiencing nutritional inadequacies may face declines while populations with sufficient nutritional resources may not.

Using pneumonia-associated pathogens in bighorn sheep (*O. canadensis*) as a model system, we sought to better understand the degree to which infection with enzootic pathogens may induce energetic trade-offs with other life functions. Respiratory pneumonia has limited populations of bighorn sheep for over a century [18], yet the complexities of the host–pathogen system impede our understanding of the disease or how to mitigate it [19]. Initial introduction of pneumonia-associated pathogens often causes mass-mortality events [19]. Some populations recover from the initial mass-mortality events, whereas others face continual declines and sometimes extirpations [20,21]. After initial outbreaks, populations often enter an enzootic stage in which adult animals can continue to carry the pathogens without experiencing clinical pneumonia, though juveniles are far more susceptible [19,22] and more commonly suffer disease and mortality [23]. Populations that are persistently infected with pneumonia-associated pathogens can experience highly variable recruitment rates, recurring epizootics or no apparent effect [24–26]. Uncertainty about the role of ecological factors, such as nutrition, contributes to a lack of understanding of the mechanisms underlying the spectrum of population responses [19,26]. Respiratory pathogens in bighorn sheep present a compelling system to assess if lurking energetic linkages associated with enzootic pathogens, particularly when not causing clinical disease [19], have insidious effects on wildlife populations.

We leveraged an individual-based dataset of repeated captures of bighorn sheep to better understand the link between energetic reserves and pathogens. The study populations were persistently infected with several pathogens associated with pneumonia [26], but adults rarely died of pneumonia. To understand fitness trade-offs that may result from interplay between energetics and infection, we evaluated the hypotheses that (H1) energetic reserves affect susceptibility or ability to clear pathogens [24] and that (H2) infection with enzootic pathogens is energetically costly [7,10]. We predicted that individuals with greater energetic reserves (i.e. fat) would carry fewer pathogens than individuals with fewer energetic reserves (H1). Additionally, we predicted that both infection or elimination of infection would reduce fat reserves (H2). The two hypotheses are not mutually exclusive, and testing both will help reveal if energetic reserves affect susceptibility to or clearance of infection (H1), if infection contributes to reduced energetic reserves (H2), or if both are occurring. Understanding the degree to which pathogens compromise individual fitness via energetic pathways and indirectly suppress populations is critical for informing conservation approaches [26].

## 2. Methods

We used repeated measures of infection status and energetic reserves in female bighorn sheep from three populations in the Greater Yellowstone Ecosystem, USA that are geographically adjacent: the Whiskey Mountain population in the Wind River Range (43.436447–109.551299); the Jackson population in the Gros Ventre Range (43.573926–110.586624); and the Upper Shoshone population in the Absaroka Range (44.420117–109.714750). Four bacterial pathogens associated with pneumonia (*M. ovipneumoniae*, leukotoxigenic *Bibersteinia trehalosi*, *Pasteurella multocida* and leukotoxigenic *Mannheimia haemolytica/glucosida*) were detected in each of the populations before and during the study [26]. *Mycoplasma ovipneumoniae* was consistently documented in most pneumonia epizootics in western North America [27], and multiple bacteria in the Pasteurellaceae family commonly also are detected in association with bighorn sheep pneumonia [28–30]. We began our study in March 2015, but did not begin testing for the full suite of Pasteurellaceae species until December 2016, and thus excluded the first year of study for all analyses that included Pasteurellaceae species.

We captured adult (4+ years old) females using helicopter net-gunning [31] and chemical immobilization. Each subsequent March and December until March 2021, we recaptured previously collared females and captured new animals to maintain sample sizes. Each animal was assigned a unique identification number. All capture and handling protocols were approved by an independent Institutional Animal Care and Use Committee at the University of Wyoming (Protocol 20180305 KM00296-03).

At each capture, we estimated ingesta-free body fat (IFBFat, hereafter, fat) using a combination of ultrasonography (5 MHz transducer; Ibex Pro, E.I. Medical Imaging, Loveland, CO, USA) to measure rump fat and body palpations to assign a body condition score [20]. Body fat is the primary energetic reserve in ungulates [7], varies seasonally, and in response to environmental conditions [32]. Over the spring and summer when forage availability is highest, sheep accumulate fat reserves. Lactation is energetically expensive and reduces the amount of fat a sheep can gain [32]. To ensure they have enough energy to survive the upcoming winter when forage is limited, sheep rely on their fat reserves to survive. Thus, bighorn sheep are capital breeders and survivors, meaning that stored energy (i.e. fat) is essential to finance reproduction and survival [10,13,33]. Each December, we determined lactation status using udder palpations to assess recruitment status [11,32]. We used horn annuli and tooth eruption to estimate the age of each sheep [34].

We collected nasal and tonsil swabs at each capture event to determine the presence of bacteria species [35]. All diagnostic tests were completed at the Wyoming Game and Fish Department Wildlife Health Laboratory using a combination of culture and PCR (see electronic supplementary material, S1, for laboratory methods). We considered a positive result in either tonsil or nasal swabs to indicate a detection of a given bacteria for the sheep at that capture event. Detection of bacterial presence was imperfect [35] and we did not differentiate between strains of bacterial species. Therefore, these data represent the minimum known bacterial species an individual was carrying during a sampling period, though it is possible our metrics of infection underestimated true infection status.

We used the number of Pasteurellaceae species (leukotoxigenic *B. trehalosi*, *P. multocida*, and leukotoxigenic *M. haemolytica/glucosida*) detected in each sheep at each capture event to quantify Pasteurellaceae richness. We used seasonal changes in fat, which requires two sequential captures of an individual (summer fat change = December fat – March fat, winter fat change = March fat – December fat), as a metric of seasonal acquisition and catabolism of energetic reserves. We also quantified changes in pathogen species detected between subsequent capture events. We defined the number of species cleared as the pathogen species that we detected in a capture event but were undetected in the subsequent capture. We defined the number of species acquired as the number of pathogen species that were undetected in a capture event but were detected in the subsequent capture. For example, if an individual tested positive for only *P. multocida* in March and only *M. haemolytica* the following December, we considered that individual to have a Pasteurellaceae richness of one at both capture events, to have cleared one Pasteurellaceae spp. (*P. multocida*) and to have acquired one Pasteurellaceae spp. (*M. haemolytica*).

Our goal was to evaluate relationships between fat and resident pathogens rather than those actively contributing to severe disease or mortality. Adult survival was generally high in our study populations, pneumonia-related mortality rare, and the pneumonia-associated pathogens were generally considered resident (i.e. the study did not take place during a pneumonia epizootic) [35,36]. Nevertheless, pathogens and fat could influence adult survival [10,33]. Our analyses of changes in fat or infection status inherently require an animal to live between seasons; mortality of individuals within the seasonal interval could underestimate estimates of relationships between pathogens and fat reserves [37,38]. Throughout the study, only six sheep died within the seasonal interval following a capture, and none of the six died of pneumonia. Thus, right-censoring likely did not bias our estimates of energetic costs of infection.

### (a) Do energetic reserves influence clearance ability or susceptibility to infection?

We used four generalized linear mixed models to determine if pre-season fat reserves (March fat for oversummer models and December fat for overwinter models) influenced seasonal acquisition or clearance of Pasteurellaceae species. The first model used oversummer acquisition of Pasteurellaceae species as the response, the second model used oversummer clearance of Pasteurellaceae, the third model used overwinter acquisition of Pasteurellaceae species and the fourth model used overwinter clearance of Pasteurellaceae. In all models, we used pre-season fat reserves as the predictor variable, and included animal identification numbers as a random intercept to account for potential pseudo-replication. Because pre-season fat reserves were the only covariate in the models, we did not conduct model selection.

We used an open occupancy model [39] to determine if pre-season fat reserves influenced acquisition or clearance of *M. ovipneumoniae* using the ‘unmarked’ package [40] in Program R [41,42]. Individuals were considered sites, and each capture event was a primary period, between which infection status can change. We did not have replicate samples for each capture, so we did not have true secondary periods, which would result in underestimation of detection probability, and therefore biased estimates of *M. ovipneumoniae* clearance or acquisition [39]. However, previous work using the same laboratory methods estimated detection probability of *M. ovipneumoniae* as 0.85 [35]. To reduce bias in the detection probability parameter, we fixed detection probability of *M. ovipneumoniae* to 0.85 [35] by creating pseudo-replication of the primary periods to create secondary periods. We replicated each individual-capture event 10 times to create secondary periods. We then adjusted 15% of the positive detections within each primary period for an individual (individual-capture events) to negative detections. The primary role of secondary periods is to estimate the detection probability parameter in occupancy models [39]; thus, our method of fixing detection probability served to produce a more accurate detection probability and reduce bias in extinction and colonization estimates.

We used population (categorical) as a site-level covariate, and fat and season (December or March) as yearly site covariates. We included population as a covariate influencing the initial probability of occupancy and to account for differences in pathogen prevalence in each population. We evaluated the influence of fat and capture event (December or March) on acquisition (i.e. colonization) and clearance (i.e. extinction) probability. We also tested for an interaction between fat and capture event, to determine if the influence of fat on acquisition or clearance of *M. ovipneumoniae* differed between seasons. We used the Akaike information criterion (AICc) adjusted for small sample size [43] to compare models with and without the interaction, and only included the interaction if it improved model fit and their influence on the response differed from zero based on 95% confidence intervals (CIs).

### (b) Does infection or clearance of infection influence energetic reserves?

We used separate generalized linear mixed models to determine if changes in fat reserves oversummer and overwinter relate to infection status and changes in infection status. We only used known predictors of seasonal fat change in our base models. Base models for oversummer fat change included March fat, reproductive status and population. Base models for overwinter fat change included December fat and winter snow depth (m) [32]. We extracted mean snow depth (m, 1 km resolution) [44] for individual home ranges following methods described in Smiley *et al.* [32]. Although prior work indicated that age influenced fat change overwinter but not oversummer [32], we included age and a quadratic effect of age in both global models because immune function and probability of infection can vary with age [22,45]. We also added infection-related variables to the base models.

The presence of pathogens, meaning testing positive for pathogens, may incur different physiological costs than new infection (acquisition of pathogens over a season) or clearance of pathogens, because the immune response required for each may differ. Thus, we developed separate models using the presence, acquisition and clearance of the pathogen species as the infection-related predictor to differentiate between possible sources of costs. Inherently, acquisition of pathogens (testing negative one season and positive the next) is collinear with the presence of pathogens (which could include either acquisition of pathogens or maintenance of pathogens, in which an animal would test positive both seasons). To identify which infection-related variable (presence, clearance or acquisition of pathogens) was most influential on changes in fat reserves, we used AICc to compare models [43] and proceeded with the variable yielding the lowest AICc value to retain in the global model.

We tested interactions between infection status and population in the global model of oversummer fat change to determine if there were different energetic costs associated with pathogens among the three study populations. We retained interaction terms if their inclusion improved model fit based on AICc and their influence on the response differed from zero based on 95% CIs. Because our models included only factors previously shown to influence seasonal changes in fat reserves [32] with the addition of infection-related and age covariates, we retained covariates from the base models and evaluated all possible combinations of age and the infection-related covariates for each model set and compared models using AICc [43,46]. All analyses were completed in Program R [41,42].

### (c) Quantifying survival and reproductive value of fat reserves

To contextualize energetic costs, we calculated the amount of energy (MJ) that sheep in various categories of infection would have available as compared with mean fat reserves of sheep with no pathogens in December. We calculated mean fat percentage of sheep in December that tested negative for all pathogens. We then subtracted the costs to fat gain of pathogens and recruitment derived from our coefficients from models of oversummer fat gain (table 1) for each individual-year. We then calculated the amount of energy (MJ) that amount of fat equated to using the energy content of fat (39.5 kJ g^−1^) as described in Stephenson *et al*. [13]. We calculated the number of days a sheep could survive on that amount of fat alone using the quotient of the energy content of fat reserves and the resting metabolic rate (291 kJ kg^−0.75^ day^−1^) [47] for the mean body mass of sheep in our study (55.9 kg) [13].

**Table 1.** Coefficients (±95% CI) from top models evaluating the influence of infection with number of Pasteurellaceae species (richness) and *M. ovipneumoniae* on change in IFBFat (percentage points) over the summer (90 observations of 52 female bighorn sheep) and winter (93 observations of 56 female bighorn sheep), 2016−2021, northwest Wyoming. Animal ID was included as a random effect. Variables with CIs that did not overlap zero are bolded.

	summer fat change		winter fat change	
predictors	estimates	95% CI	estimates	95% CI
**(intercept)**	**20.69**	**(16.62, 24.76)**	**4.03**	**(2.19, 5.86)**
**pre-season fat (%)**	**−1.06**	**(−1.31, –0.80)**	**−0.55**	**(−0.65, –0.44)**
**Pasteurellaceae richness**	**−1.41**	**(−2.58, –0.24)**	**−0.99**	**(−1.55, –0.43)**
***M. ovipneumoniae***	**−2.92**	**(−5.24, –0.60)**		
population [Jackson]	−1.41	(−4.07, 1.26)		
**population [Whiskey Mountain]**	**−5.56**	**(−8.29, –2.83)**		
**recruitment**	**−5.28**	**(−6.86, –3.71)**		
**snow depth (m)**			**−2.71**	**(−4.34, –1.08)**

To directly assess the influence of fat reserves on fitness metrics, we modelled the influence of fat reserves on the probability of overwinter survival and the probability of pregnancy. We used Cox proportional hazard models to assess overwinter survival of bighorn sheep for five months following capture in December. We used monthly encounter histories and included fat in December, age, age^2^ and population as a categorical covariate. We censored animals that died during or after 3 days of capture [31] or had collar failures between December and May. We included population as a categorical covariate and animal ID as a random intercept. We used a logistic regression to determine the influence of December fat on probability of pregnancy the following March. We included December fat, and population as a categorical covariate. We were not able to include a random effect in the model because of a small sample of non-pregnant animals.

## 3. Results

Between 2015 and 2021, we captured 128 total individuals in 470 total captures. We obtained 90 oversummer transitions (i.e. sequential capture events from March to December of the same year for one individual) of 52 sheep and 93 overwinter transitions (i.e. sequential capture events from December to March of the following year for one individual) of 56 sheep within the three populations (electronic supplementary material, table S1). Average fat for all sheep was 13.92% (s.e. = 0.46) in December and 8.60% (s.e. = 0.31) in March. Age of sheep ranged from 4 to 13 years old (mean 7.80 years, s.e. = 0.15).

### (a) Do energetic reserves influence ability to clear or susceptibility to infection?

**Figure 1.**
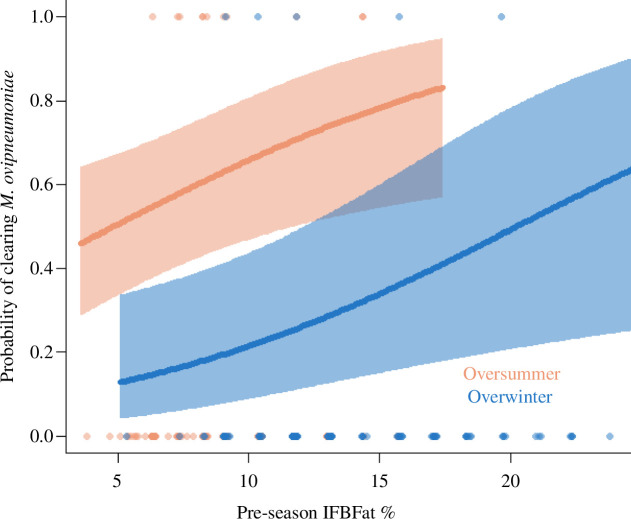
Relationship between IFBFat % at the beginning of a season and the probability of clearing *M. ovipneumoniae* over the subsequent season in bighorn sheep in northwest Wyoming between 2015 and 2021. We used open occupancy models with population included as a covariate influencing the initial probability of occupancy. Individuals were considered sites and detection probability was fixed to 0.85.

Pre-season energetic reserves influenced the probability of clearing *M. ovipneumoniae* but did not influence the probability of acquiring *M. ovipneumoniae* nor change in infection status (clearance or acquisition) for Pasteurellaceae species. In the models of acquisition and clearance of Pasteurellaceae for both oversummer and overwinter, CIs of the effect of fat on infection status overlapped zero (electronic supplementary material, table S2). In the models of acquisition and clearance of *M. ovipneumoniae*, the interaction between season and fat overlapped zero and increased AICc compared with the model without the interaction (ΔAICc = 4.33), so we did not include the interaction in the final model. Fat influenced the probability of clearing *M. ovipneumoniae* (*β* = 0.13, 95% CI = 0.02, 0.23) and sheep were more likely to clear *M. ovipneumoniae* over summer (*β* = 1.95, 95% CI = 0.56, 3.34; electronic supplementary material, table S3; figure 1).

### (b) Does infection and clearance of infection influence energetic reserves?

Global models of energetic costs of infection that included the presence of *M. ovipneumoniae* at the end of the season outperformed the global model using *M. ovipneumoniae* acquisition (ΔAICc = 3.63) or clearance (ΔAICc = 7.52) over summer and marginally over winter (ΔAICc between acquisition, clearance and presence <1). Models using Pasteurellaceae richness at the end of a season outperformed models using Pasteurellaceae species acquired (ΔAICc = 2.25) or cleared (ΔAICc = 1.67) over summer and acquired (ΔAICc = 8.57) or cleared (ΔAICc = 6.73) over winter. Therefore, to evaluate the energetic costs of infection, we included *M. ovipneumoniae* presence and Pasteurellaceae richness at the end of the season for summer and winter models.

**Figure 2.**
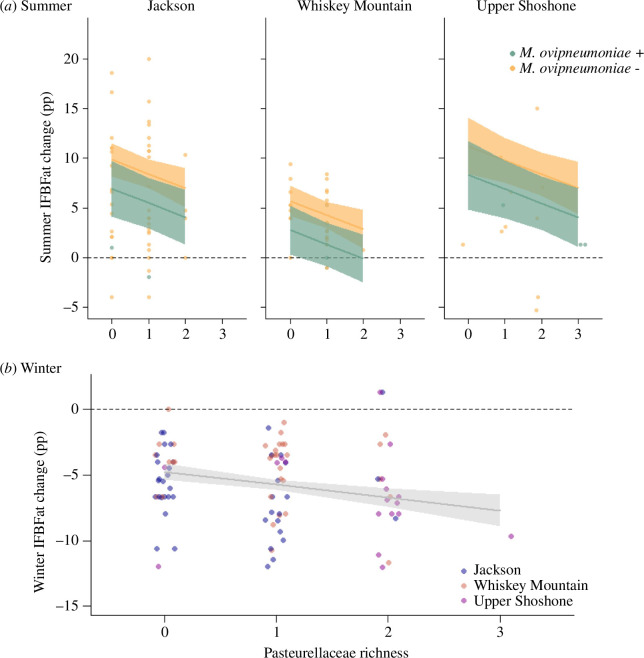
Pathogens decrease the amount of fat that sheep gain over summer and increase the amount of fat that sheep lose over winter. The panels display the relationship between pathogens (*M. ovipneumoniae* and Pasteurellaceae richness; number of Pasteurellaceae species detected, *P. multocida*, leukotoxigenic *B. trehalosi*, and leukotoxogenic *M. haemolytica/glucosida*) and change in IFBFat (percentage points) oversummer (*a*) and overwinter (*b*) in bighorn sheep in northwest Wyoming between 2016 and 2021. Raw data are included, though model predictions also account for other factors that influence seasonal change in fat (summer: March IFBFat, recruitment status; winter: December IFBFat, snow depth), and animal ID was included as a random effect.

The top model for oversummer change in fat reserves included the presence of *M. ovipneumoniae* and Pasteurellaceae richness at the end of season, March fat, recruitment status and population (table 1; ΔAICc from base model = 9.45). During winter, the top model included Pasteurellaceae richness at the end of season, December fat and snow depth (table 1; ΔAICc from base model = 9.91). Pasteurellaceae richness resulted in decreased fat gain over summer of 1.41 percentage points for each species. Over winter, Pasteurellaceae richness increased fat loss of 0.99 percentage points for each species (figure 2). Infection with *M. ovipneumoniae* was associated with reduced fat gain by 2.92 percentage points over the summer (table 1; figure 2*a*) but did not influence fat change over winter (table 2; figure 2*b*).

**Table 2.** Percentage points (pp) of IFBFat translated to available energy (MJ) and the number of days a bighorn sheep could survive on that amount alone with no additional energetic intake. Costs to fat were derived from model outputs of costs to fat gain over the summer. We subtracted the costs to fat gain of various infection and reproductive statuses from the mean amount of fat of uninfected sheep (15.35%) observed in our study with 90 individual-years of 53 bighorn in northwest Wyoming from 2016 to 2021. *N* represents the number of individual-years that incurred the associated cost observed in the study.

category of energetic cost	*N*	cost to fat (pp)	fat (IFBFat %; December)	energy (MJ)	days surviving on fat alone
no pathogens	27	0.00	15.35	301.89	45
1 Pasteurellaceae spp.	42	1.41	13.94	271.25	41
*M. ovipneumoniae* + 1 Pasteurellaceae spp.	8	4.33	11.02	207.81	31
*M. ovipneumoniae* + 3 Pasteurellaceae spp.	2	7.15	8.20	146.55	22
recruited, no pathogens	35	5.28	10.07	187.17	28

### (c) Quantifying survival and reproductive value of fat reserves

**Figure 3.**
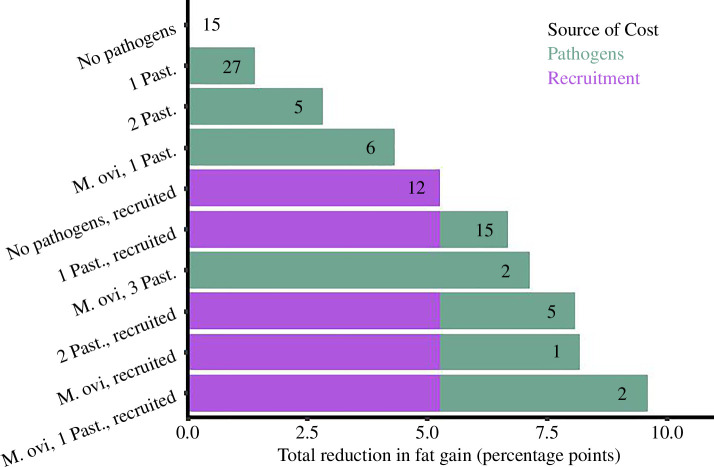
Reduced fat gain over summer associated with infection with *M. ovipneumoniae* (*M. ovi*), Pasteurellaceae species (Past.; *M. haemolytica*, *P. multocida*, and *B. trehalosi*), and recruitment observed in a study of 90 bighorn sheep in northwest Wyoming, 2016−2021. Costs to fat gain were calculated by applying coefficients from models of fat gain over summer to the infection status and recruitment status for each individual. Total costs are calculated by adding the costs associated with pathogens to the costs associated with recruitment. Black numbers within the bars represent the sample size of individual-years observed in each category. For example, 12 sheep recruited over the summer and were not carrying any pathogens.

Based on available energy, infection reduced the number of days a sheep could survive on fat alone by up to 23 days (table 2) when compared with an uninfected sheep (no pathogens, mean body fat = 15.35%), which have the energy reserves to survive for 45 days without any food intake. Reproduction reduces the number of days a sheep could survive on fat alone by 17 days (table 2). In our study, 10 sheep experienced costs to fat gain associated with pathogens that were equal to or greater than the costs associated with lactation over summer (figure 3).

Fat gain over the summer was correlated with fat in December (correlation coefficient = 0.83), which we used to investigate relationships between fat and fitness (probability of overwinter survival and pregnancy) in bighorn sheep. During our study, 38 collared sheep died, but we only had fat measurements from the preceding December for six of them. Of all mortalities of which we were able to identify the cause, only one died of pneumonia, and we had not captured her for over a year before death, and thus it was not included in the analysis that year. During the study, 10 animals for which we had fat measurements from December were not pregnant the following March. Age did not influence probability of overwinter survival (electronic supplementary material, table S4) and overwinter survival nor pregnancy rates differed between populations (electronic supplementary material, tables S4 and S5). Fatter animals were more likely to survive through winter and be pregnant in the spring than animals with less fat (electronic supplementary material, figures S1 and S2). A reduction in 3 percentage points of fat in December (from 14% to 11%) reduced the probability of overwinter survival from 0.79 to 0.46 and the probability of pregnancy from 0.93 to 0.78 on average, though CIs are relatively large given small sample sizes (electronic supplementary material, tables S4 and S5).

## 4. Discussion

Host response to pathogens is a fundamental life-history component that affects energetic requirements for maintenance and survival [48]. Fundamentally, when energetic resources are limited, there are trade-offs in allocation to competing life functions; for adults, allocation to maintenance is prioritized over reproduction [49]. We investigated relationships between energetics and pathogens to better understand if the presence of resident pathogens may indirectly influence population dynamics via energetic pathways. Fat reserves influenced the ability of bighorn sheep to clear *M. ovipneumoniae*, and infection with M. *ovipneumoniae* and Pasteurellaceae species reduced fat reserves. Bighorn sheep that were fatter were more likely to clear *M. ovipneumoniae* (figure 1), the primary pneumonia-causing pathogen, than animals with less fat. Over summer, reduced fat gain associated with infection approached and, in some instances, exceeded the cost of reproduction to fat (figure 3). Independent of the disease-causing potential of the pathogens, energetic burden of pathogens reduced the amount of energy available for survival by up to 23 days if sheep were surviving on fat alone (table 2). In bighorn sheep, infection reduced fat reserves, a vital resource that not only is essential for survival and reproduction, but also aids pathogen clearance.

**Figure 4.**
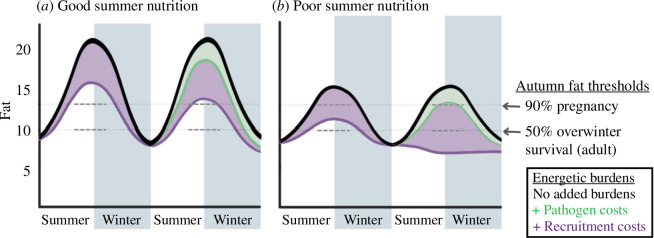
Conceptualization of energetics in the context of reproductive and pathogen-related costs for bighorn sheep. Estimates of thresholds for overwinter survival, pregnancy, seasonal fat changes and costs to fat in the figure are based on data from a study of bighorn sheep in northwest Wyoming between 2016 and 2021. Costs of pathogens to fat displayed in figure represent the mean costs incurred in our study (2.2 percentage points), but costs could exceed 7 percentage points for animals carrying several pathogen species (*M. ovipneumoniae* and Pasteurellaceae species). Energy obtained over the summer when food is abundant is stored as fat reserves that are used to finance survival costs over winter when there is an energetic deficit. For example, sheep need to have approximately 10.5% fat in autumn to have a 50% chance of surviving the winter and 14% fat to have a 90% chance of pregnancy the following spring. Raising offspring over the summer and coping with pathogens incur energetic costs, thereby lessening the amount of fat reserves an animal can accumulate over the summer. Because coping with pathogens is necessary for maintenance and survival, animals are not likely able to regulate the costs associated with coping with pathogens. If the individual is at risk of not accumulating enough fat reserves to ensure survival over winter, they may need to sacrifice energetic allocation to recruiting offspring. In a population with robust summer ranges and sufficient nutritional availability (*a*), sheep usually can gain sufficient fat over summer, even if they recruit and are carrying pathogens. In contrast, in a population with poor summer ranges (*b*), sheep likely cannot endure the costs of pathogens, recruit and gain sufficient fat reserves to ensure survival over the winter. Indeed, recruiting offspring and coping with pathogens can result in loss of fat over the summer, thereby dramatically lessening the probability of survival.

Our study provides a basis for understanding how immune challenges can have substantial, long-term costs and result in trade-offs with energetic allocation towards reproduction in favour of survival. Across taxa, energetic requirements of immune responses [50] can result in elevated metabolic rate [51–53], suggesting fine-scale energetic costs. Measurable changes in body fat for bighorn sheep indicate that fine-scale costs of infection accumulate to detectable reductions in energetic reserves across seasons [54]. Fat reserves directly influence fitness (figure 4; electronic supplementary material, figures S1, S2) and population growth in ungulates [7,10,11,13]. Thus, the reduction in energetic reserves because of infection may have consequences for fitness and ultimately, influence population dynamics.

Life-history characteristics of the host shape its response to infection [55], and immune responses can have trade-offs with other life-history components [56–58]. In long-lived iteroparous species, maintenance of immune function is essential to survival and future reproductive opportunities, and therefore should be favoured over allocation of energy to current reproduction. For bighorn sheep, which are capital breeders and survivors with conservative reproductive tactics [59,60], excess energetic costs are transferred to offspring [60,61]. When resources were limited, juveniles experienced a reduction in mass gain, thereby reducing the probability of offspring survival over winter [60,61]. Moreover, sheep with minimal fat reserves were less likely to clear *M. ovipneumoniae* (figure 1), a pathogen that can cause disease and mortality in juveniles [19,62]. Thus, mothers with insufficient fat have reduced probability of reproductive success via reduced allocation and increased pathogen exposure. Indeed, in our study populations, though adult survival and pregnancy rates were high (electronic supplementary material, figures S1 and S2), recruitment rates often were lower and more variable, ranging from 20% to 57% [32]. Not only are juveniles more susceptible to disease, but their survival may be further compromised as mothers prioritize energy allocation to contending with the energetic burden of infection.

The disparity in population dynamics of bighorn sheep infected with pneumonia has been a conundrum for over a century [18]. Juvenile survival, which often is highly variable across years [25], is the primary driver of slowed population growth or decline [19]. Bacterial strain may contribute to the disparity in the form of variable pathogenicity [24] but is insufficient to explain the large disparity in performance among populations that are persistently infected with pneumonia-associated pathogens. Across taxa, nutrition largely underpins population dynamics [8,9,63], and chronically infected populations are no exception. Our three study populations are genetically distinct [64], and although the genetics of both the host and pathogen can influence outcomes of infection [65], the populations did not differ in the energetic costs associated with pathogens. Among the populations in our study, a disparity in nutritional dynamics is co-occurrent with forage availability, recruitment rates and population trends [32,36]. Our study provides mechanistic evidence that infection with resident pathogens compromises the maintenance of energetic reserves (figure 2), which are necessary for fitness components, including clearance of pathogens (figure 1), reproduction (electronic supplementary material, figure S2) and survival (figure 4; electronic supplementary material, figure S1). Consequently, high energetic burdens associated with infection may disproportionally affect populations with limited resource availability (figure 4).

## 5. Conclusions

The direct effect of pathogens on their wildlife hosts is obvious, yet there is growing evidence that pathogens may indirectly underpin demographic rates. Many well-studied pathogens have clear mechanisms for influencing demographic rates in ungulates via mortality from disease [66,67], reduced body condition because of intestinal parasites [68–70] and reduced reproductive success because of abortion-causing bacteria [4,71]. Resident respiratory bacteria, which are common across taxa [72,73], have less obvious links to fitness in the absence of clinical disease. Nevertheless, the pathogens in our study were associated with substantial reductions in energy stores for bighorn sheep (figure 2) that hinder their ability to clear pathogens and inevitably hold downstream consequences for fitness. Indirect effects of pathogens, therefore, play a role in suppressed recovery or population growth via energetic trade-offs. Overlooking the interplay between nutrition and pathogens may lead to conservation strategies which ignore the underlying factors that hinder population growth.

Insufficient food resources and high energetic burdens associated with infection may underlie poor population performance for bighorn sheep. Though the role of population density in pathogen transmission is potentially inconsequential in bighorn sheep–pneumonia systems [64,74], population density certainly influences forage availability, animal condition and reproductive success [61,75,76]. Our study reveals linkages between nutrition and pathogens which provide context for understanding variable dynamics of bighorn sheep populations and reinforces emphasis on population or habitat management, in concert with preventing pathogen spillover or reducing prevalence [74], as conservation strategies.

## Data Availability

Data used in the study and code used for statistical analyses are archived in Dryad [42]. Supplementary material is available online [77].
